# Unique glycosignature for intervertebral disc and articular cartilage cells and tissues in immaturity and maturity

**DOI:** 10.1038/srep23062

**Published:** 2016-03-11

**Authors:** E. C. Collin, M. Kilcoyne, S. J. White, S. Grad, M. Alini, L. Joshi, A. S. Pandit

**Affiliations:** 1 Center for Research in Medical Devices (CÚRAM), National University of Ireland Galway, Ireland; 2 Microbiology, School of Natural Sciences, National University of Ireland Galway, Ireland; 3 Glycoscience Group, National University of Ireland Galway, Ireland; 4 AO Research Institute, Davos, Switzerland

## Abstract

In this study, on/off markers for intervertebral disc (IVD) and articular cartilage (AC) cells (chondrocytes) and distinct glycoprofiles of cell and tissue-types were identified from immaturity to maturity. Three and eleven month-old ovine IVD and AC tissues were histochemically profiled with a panel of lectins and antibodies. Relationships between tissue and cell types were analysed by hierarchical clustering. Chondroitin sulfate (CS) composition of annulus fibrosus (AF), nucleus pulposus (NP) and AC tissues was determined by HPLC analysis. Clear on/off cell type markers were identified, which enabled the discrimination of chondrocytes, AF and NP cells. AF and NP cells were distinguishable using MAA, SNA-I, SBA and WFA lectins, which bound to both NP cells and chondrocytes but not AF cells. Chondrocytes were distinguished from NP and AF cells with a specific binding of LTA and PNA lectins to chondrocytes. Each tissue showed a unique CS composition with a distinct switch in sulfation pattern in AF and NP tissues upon disc maturity while cartilage maintained the same sulfation pattern over time. In conclusion, distinct glycoprofiles for cell and tissue-types across age groups were identified in addition to altered CS composition and sulfation patterns for tissue types upon maturity.

Neck and low back pain, affecting 80% of the population over its lifetime^1^, is strongly associated with ageing and degeneration of the intervertebral discs (IVD)^2^. The IVD provide flexibility and mobility to the spine. During degeneration, ageing or injury, this organ loses its flexibility and its structural integrity associated with an inherent inability to self-repair^2^,^3^. IVD is composed of the nucleus pulposus (NP), a highly hydrated gelatinous tissue, the annulus fibrosus (AF), an elastic fibrous tissue surrounding the NP, and the cartilage end-plate (CEP), capping the AF and NP on both sides of the vertebrae^2^,^4^. At a healthy state, IVD cells comprise only 1% of the IVD tissue volume but are essential to maintain tissue health and function^5^. Different cell types can be found within the IVD tissue: AF cells, chondrocytes and NP cells^6^. Recent studies also suggest the presence of progenitor cells^7^,^8^,^9^ and the maintenance of remaining notochordal cells^10^ within this tissue. NP cells are distinct from chondrocytes by the ratio of extracellular matrix (ECM) that they produce and their origin, attributed notochordal for NP cells while mesenchymal for chondrocytes^5^,^11^,^12^,^13^. During degeneration, the IVD cell population decreases greatly starting with the loss of notochordal cells^3^. ECM composition is severely affected, notably with proteoglycan (PG) depletion and altered distribution^2^,^14^,^15^

NP and AF ECM composition differs according to tissue function and status, *i.e.* health, disease, maturity and degeneration^14^. The NP is mainly composed of type II collagen, aggrecan and hyaluronic acid (HA) and AF of type I collagen and fibronectin^2^,^11^. Other collagens types^6^,^14^, connective proteins, such as laminin and elastin^6^, and other PGs such as decorin, biglycan, versican, lumican, and fibromodulin^16^ expressed in both tissues at varying concentrations. ECM PG composition and distribution changes during growth and development^16^. Current IVD regenerative strategies include replenishing the ECM with a scaffold and/or delivery of mesenchymal stem cells (MSCs) for tissue repopulation and regeneration^14^,^16^. To produce the right ECM for IVD regeneration it is important that implanted MSCs differentiate towards the correct cell type. Currently, MSC differentiation is monitored by a ratiometric expression of genes and proteins in the tissue^13^, e.g. cytokeratin-19^17^, FOXF1^18^ and CA-12^18^,^19^ genes more highly expressed in NP cells than in chondrocytes and AF cells. Recent tissue engineering studies have mainly used a small panel of chondrogenic differentiation markers including type II collagen, aggrecan, and Sox9^20^,^21^. However, although chondrocytes and NP cells secrete compositionally similar ECM, their morphology and their ECM at a quantitative level are different^18^. Indeed, Mwale *et al.* have reported a ratio 27:1 of GAG:collagen content for NP tissue as opposed to a ratio of 2:1 for hyaline cartilage^22^. Thus, the identification of clear “on/off” cell surface expressed markers to distinguish between chondrocytes, NP and AF cells is important for tissue regenerative strategies^13^.

Glycocalyx coat of cells are cell specific and can be used as cell markers, e.g. SSEA-3 and -4 glycolipids used to identify human embryonic stem cells^23^,^24^. Cell surface glycosylation alters temporally and spatially during differentiation, development and disease^24^,^25^,^26^ and reflects the cell phenotypic and tissue biological status^27^. Glycosylation has numerous biological roles, including cellular homing and trafficking, signalling, cell-cell and cell-ECM communication and adhesion^24^,^26^,^28^. Glycans exert their biological effects *in vivo via* lectins, carbohydrate-binding proteins^24^,^26^. As glycan cell surface and ECM composition is altered depending on the tissue and cell type and its status, correct IVD ECM composition and cell health are inextricably linked. Thus, the cell surface glycosylation of healthy immature and mature IVD cells can provide cell type markers and biological status indicators.

In addition to PG variation, the composition, length, sulfation pattern and distribution of glycosaminoglycans (GAGs) is altered during IVD growth, development and ageing^16^,^29^,^30^. ECM and cell-associated GAG in IVD, of which chondroitin sulfate (CS), play important biological roles in addition to their physicochemical properties allowing a high tissue hydration^16^,^29^. The specific sulfation pattern of CS and heparan sulfate (HS) governs and modulates binding to bioactive molecules, including growth factors, to precipitate biological signalling, cell production and ECM interactions^29^,^30^. CS structural changes during IVD maturation are critical to maintain tissue at a healthy stage and a better understanding would enable more effective tissue engineering approaches and provide another metric for healthy IVD ECM^18^.

Plant lectins are widely used for carbohydrate detection in cells and tissues^24^,^31^. In this study a panel of lectins and carbohydrate-specific antibodies was used to glycoprofile immature and mature IVD and cartilage tissues. CS quantity and sulfation pattern in each tissue was also analysed by high performance liquid chromatography (HPLC). Several on/off glycosylation-based biomarkers were identified to distinguish the IVD and cartilage cell types. The hypotheses of this study were, firstly, that IVD and cartilage tissues could be selectively distinguished based on glyco-biomarkers at cell and ECM levels, secondly, that change in glycan expression and structure occur on the cell surfaces and in the ECM with maturation. Distinct glycoprofiles for cell and tissue types across age groups were determined in addition to discrete CS composition and sulfation patterns for tissue types which were altered upon IVD maturity.

## Results

### Glycoprofiling of immature and mature IVD and cartilage tissue

Bovine and ovine IVD resemble human IVD in the occurrence of notochordal cells in immaturity (Fig. S1)^32^, mechanical properties^33^, biochemical composition^3^, and gene expression^34^. Therefore ovine lumbar IVDs (L4-L5) and cartilage tissues were used to identify cell type biomarkers. A library of lectins and carbohydrate-specific antibodies was selected based on typical mammalian-type glycosylation.

### Fucosylation

**Figure 1:**
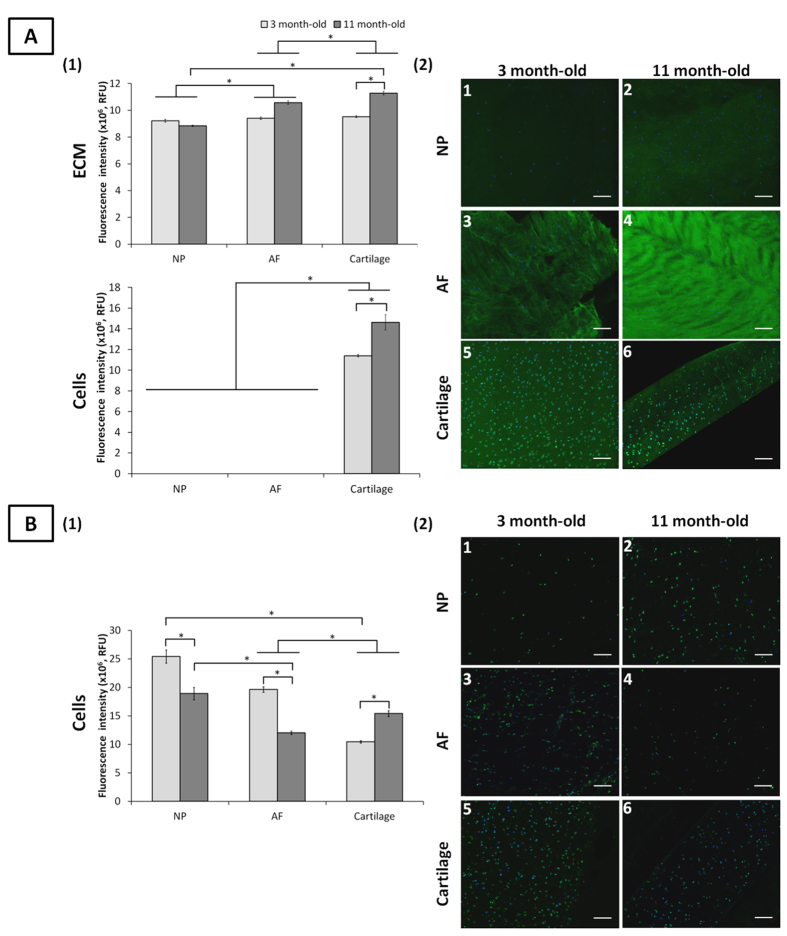
Fucosylated motifs detected by LTA lectin and Le^b^ antibody staining in ovine NP, AF and cartilage tissues at three and 11 months. Quantification of LTA lectin (A1) and Le^b^ antibody (B1) binding to the ECM and onto the cells of NP, AF and cartilage tissues. Data were normalised to surface area and represented as mean ± standard error of the mean (n = 5). *denotes significant differences between the different groups at *p *< 0.05. Representative fluorescent images of the stained fucosylated motifs detected by LTA lectin (A2) and Le^b^ antibody (B2) binding in NP, AF, and cartilage tissues. Fucosylated motifs and nuclei are stained in green and blue, respectively. Scale bar = 100 μm.

The fucose (Fuc) binding lectins UEA-I, AAA, and LTA and the anti-Le^b^ antibody have distinct and overlapping linkage specificities (Table S1). Tissue binding was unique for each lectin (Figs 1 and S2). Le^b^ was detected on all cells but was absent in all ECM for both age groups (Fig. 1B). With ageing, a significant decrease in Le^b^ expression on NP and AF cells (25.6% and 38.7% reduction, respectively) was noted while its expression increased on chondrocytes. Overall Le^b^ expression was higher for NP cells than AF cells and chondrocytes, independent of age. Similar cell type binding profiles were observed for UEA-I and AAA (Fig. S2). For both lectins, immature NP and AF cells had higher binding intensity than chondrocytes. NP and AF cell binding decreased significantly over time (except for AAA binding to AF cells), with increased binding to chondrocytes. LTA only bound to chondrocytes, independent of age (Fig. 1A) with an increased intensity upon maturity (22.1% higher, *p *< 0.05). The preferential binding of LTA is the motif α-(1→6)-linked Fuc on the chitobiose core of *N*-linked oligosaccharides and to Le^x^^35^. It also recognises poorly α-(1→2)-linked Fuc (the H-antigen) which explains the lack of binding to NP and AF cells, both recognised by UEA-I and AAA.

The H-antigen, core α-(1→6)-linked Fuc and Le^x^ structures were present in all ECM tissues as indicated by LTA, UEA-I and AAA. Cartilage ECM had increased binding of LTA and UEA-I upon maturity with no significant differences observed for NP and AF ECM (Figs 1 and S2). Overall, LTA bound to cartilage ECM with greater intensity compared to NP and AF ECM, independent of age. No significant differences of UEA-I and AAA binding were noted between maturity and immaturity, indicating a constant H-antigen expression in all ECM tissues over time.

### Sialylation

**Figure 2:**
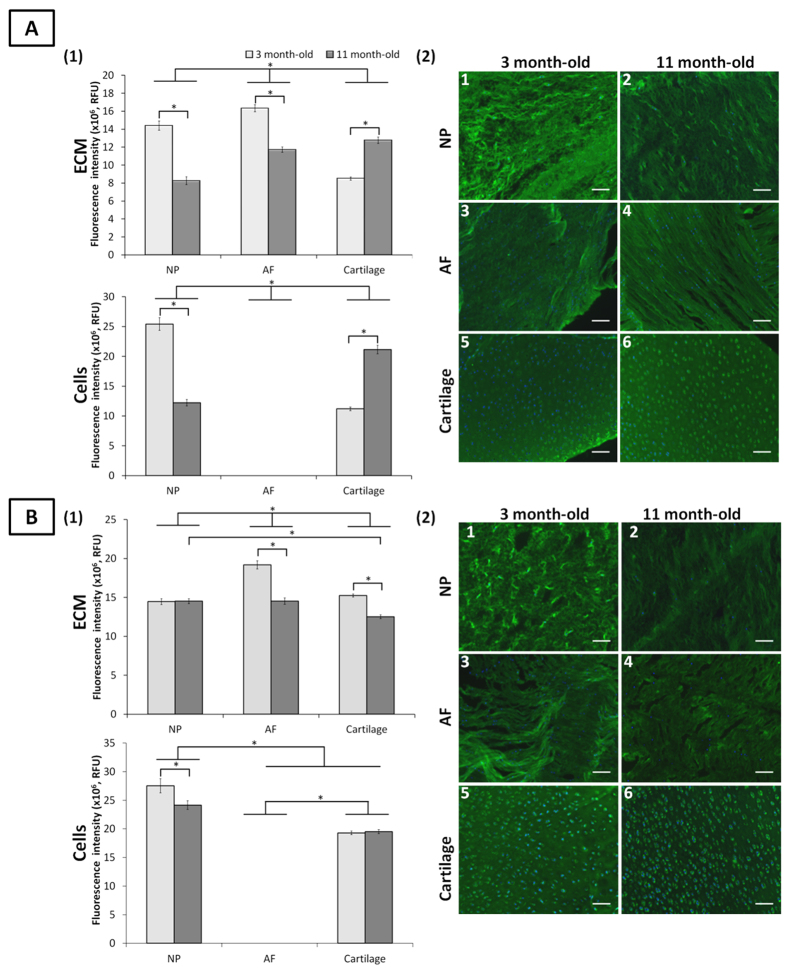
Sialylated motifs detected by SNA-I and MAA lectin staining in ovine NP, AF and cartilage tissues at three and 11 months. Quantification of SNA-I (A1) and MAA (B1) lectins binding to the ECM and onto the cells of NP, AF and cartilage tissues. Data were normalised to surface area and represented as mean ± standard error of the mean (n = 5). *denotes significant differences between the different groups at *p *< 0.05. Representative fluorescent images of the stained sialylated motifs detected by SNA-I (A2) and MAA (B2) lectins binding in NP, AF, and cartilage tissues. Sialylated motifs and nuclei are stained in green and blue, respectively. Scale bar = 100 μm.

MAA and SNA-I bind to α-(2→3)-linked sialic acid and α-(2→6)-linked sialic acid, respectively (Table S1). Neither lectin bound to AF cells independent to the age-group, which indicated a lack of sialic acid on these cells. Both lectins bound to chondrocytes and NP cells independent of age. An inversion of α-(2→6)-linked sialic acid expression on NP cells and chondrocytes was observed with age with a higher binding of SNA-I on NP cells at immaturity and a higher binding on chondrocytes at maturity (Fig. 2A). Overall, MAA binding to NP cells was higher than to chondrocytes at both age groups (Fig. 2B). No change of binding on chondrocytes was observed upon maturity which indicated constant α-(2→3)-linked sialic acid expression.

Both lectins bound to all ECM tissues. SNA-I binding was higher in immature NP and AF ECM compared to immature cartilage. The inverse trend was observed at maturity with higher binding in mature cartilage compared to mature NP and AF ECM. For both age-groups, AF ECM expressed more α-(2→6)-linked sialic acid than NP ECM (Fig. 2A). The difference in MAA binding between age groups and tissue types was less pronounced than for SNA-I (Fig. 2B).

### Galactosylation

**Figure 3:**
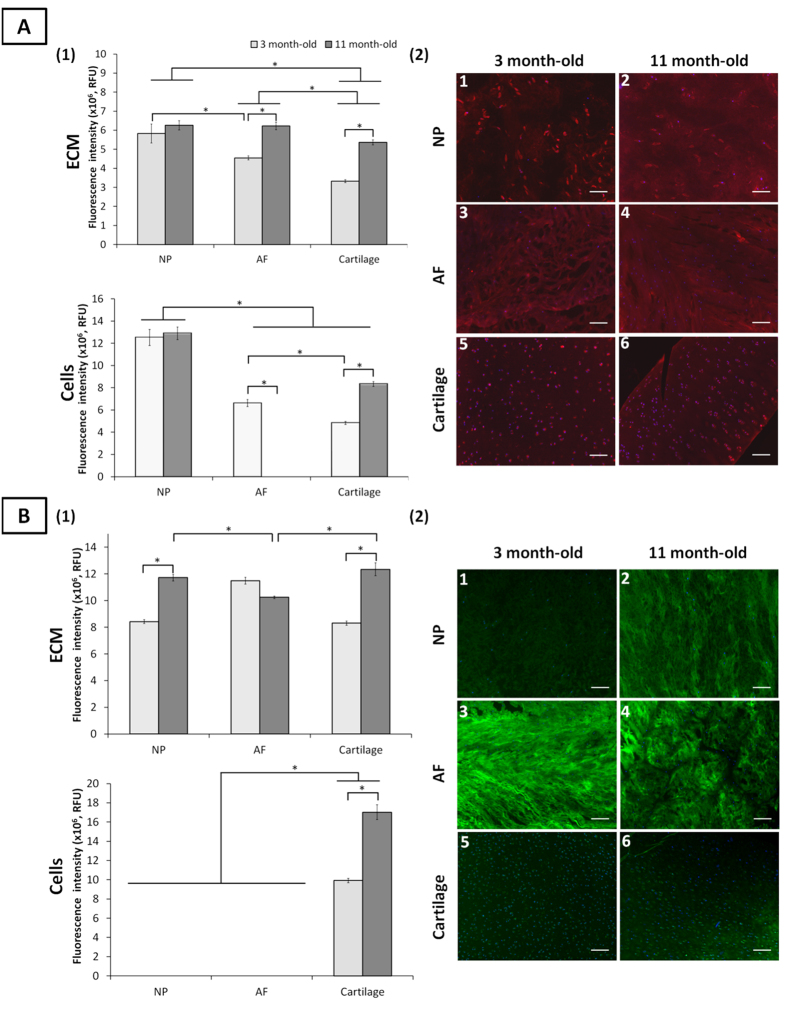
Mannosylated and galactosylated motifs detected by Con A and PNA lectin staining, respectively, in ovine NP, AF and cartilage tissues at three and 11 months. Quantification of Con A (A1) and PNA (B1) lectins binding to the ECM and onto the cells of NP, AF and cartilage tissues. Data were normalised to surface area and represented as mean ± standard error of the mean (n = 5). *denotes significant differences between the different groups at *p *< 0.05. Representative fluorescent images of the stained motifs detected by Con A (A2) and PNA (B2) lectins binding in NP, AF, and cartilage tissues. Mannosylated motifs and nuclei are stained in red and blue, respectively. Galactosylated motifs and nuclei are stained in green and blue, respectively. Scale bar = 100 μm.

Both PNA and Jacalin lectins bind to Gal-β-(1→3)-GalNAc (T-antigen) and to terminal β-linked galactose (Gal). Jacalin also recognises sialic acid terminated T-antigen (Table S1). PNA bound only to chondrocytes while Jacalin bound to all cell types, independent of age (Figs 3B and S3B). A lack of MAA and SNA-I binding to AF cells indicated the absence of cell surface siaylation (Fig. 2), which implies the presence of sulfated rather than sialylated T-antigen on AF cells^35^. PNA binding on chondrocytes increased upon maturity (41.7%). Overall Jacalin binding was greatest on immature NP cells, which decreased upon maturity (13.6%) to the same intensity than that of AF cells.

Both lectins stained all ECM tissues with a constant binding intensity, irrespective of age. However, non-sialylated T-antigen expression, as indicated by PNA binding, varied across age groups with an increased intensity over time in both NP and cartilage ECM (32.7% and 28.1%, respectively) and decreased in AF ECM.

Terminal α-galactose motifs, detected by the GS-I-B4 (Table 1) bound on the surface of both age-groups (Fig. S3). An increase of binding intensity was noted for chondrocytes and NP cells upon maturity while the intensity decreased onto AF cells (33%). A comparable binding pattern was observed at an ECM level. The staining revealed the presence of two cell subpopulations within the AF tissue.

### High mannose type glycosylation

Con A lectin preferentially binds to mannose (Man) motif and generally indicates high mannose type *N*-linked glycosylation on glycoproteins^18^. Con A stained all immature cell types while only NP cells and chondrocytes at maturity. An iterative decrease of binding was observed between the three cell types from NP cells to chondrocytes in immature tissue (Fig. 3A). Upon maturity, a markedly increase of binding was noticed for chondrocytes (42.0%).

Con A bound to all ECM tissues. For immature tissues, an iterative decrease in binding was observed from NP to cartilage ECM. Upon maturation, the binding intensity increased for all tissues, with AF and NP ECM reaching a similar binding intensity both greater than cartilage ECM.

### Terminal GalNAc

**Figure 4:**
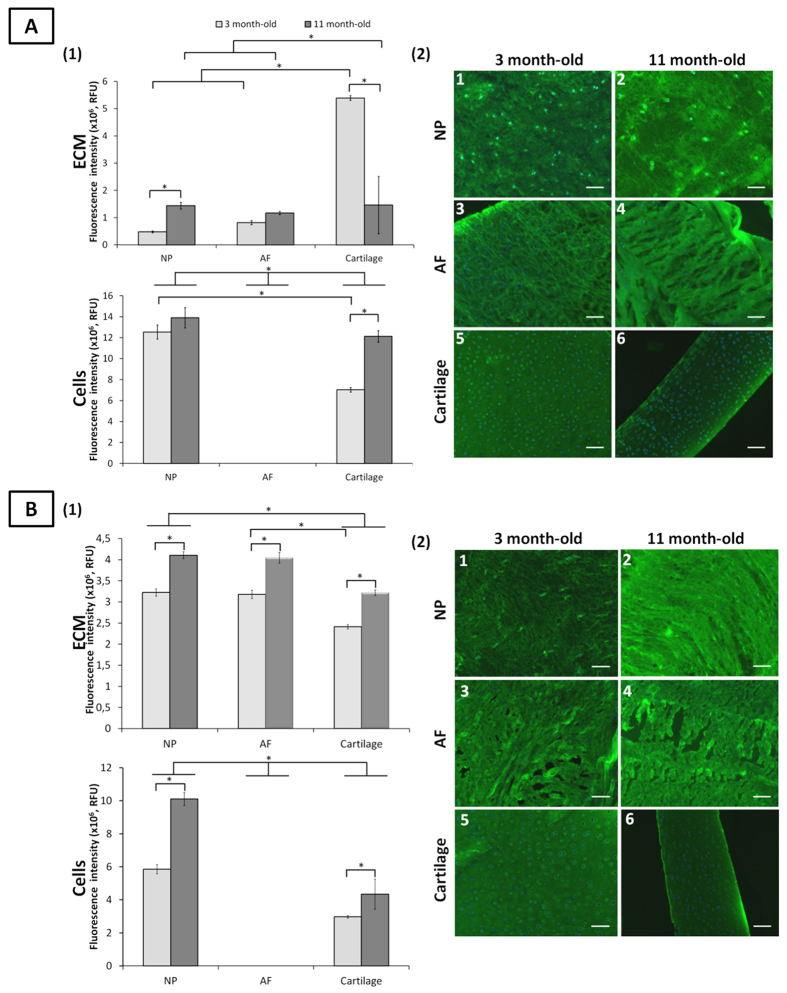
Terminal GalNAc motifs detected by SBA and WFA lectin staining in ovine NP, AF and cartilage tissues at three and 11 months. Quantification of SBA (A1) and WFA (B1) lectins binding to the ECM and onto the cells of NP, AF and cartilage tissues. Data were normalised to surface area and represented as mean ± standard error of the mean (n = 5). *denotes significant differences between the different groups at *p *< 0.05. Representative fluorescent images of the stained motifs detected by SBA (A2) and WFA (B2) lectins binding in NP, AF, and cartilage tissues. Terminal GalNAc motifs and nuclei are stained in green and blue, respectively. Scale bar = 100 μm.

Both SBA and WFA lectins have a strong binding affinity for terminal *N*-acetylgalactosamine (GalNAc) and recognise galactose motifs contained notably in chondroitin sulfates^36^. No binding of SBA and WFA were observed onto AF cells at both ages (Fig. 4). Binding of both lectins on chondrocyte and NP cells increased over time, with greatest intensity for NP cells for both age groups.

Both lectins bound to all ECM tissues. The binding intensity of SBA was greatest in cartilage ECM which drastically decreased with ageing (92.2%) indicating a loss of terminal GalNAc motifs. SBA binding increased for NP ECM upon maturity (66.5%). WFA binding increased upon maturity for all ECM tissues (Fig. 4B).

### CS analysis in immature and mature IVD and cartilage tissue

Anti-CS antibodies (anti-CS-56 and anti-C6S) recognised epitopes on all ECM tissues while no epitope was detected on cell surface. Overall, anti-CS-56 antibody binding increased with maturity with a greatest in cartilage ECM at both age groups (Fig. S4M). A significant decrease in Binding intensity of anti-C6S antibody.

**Figure 5:**
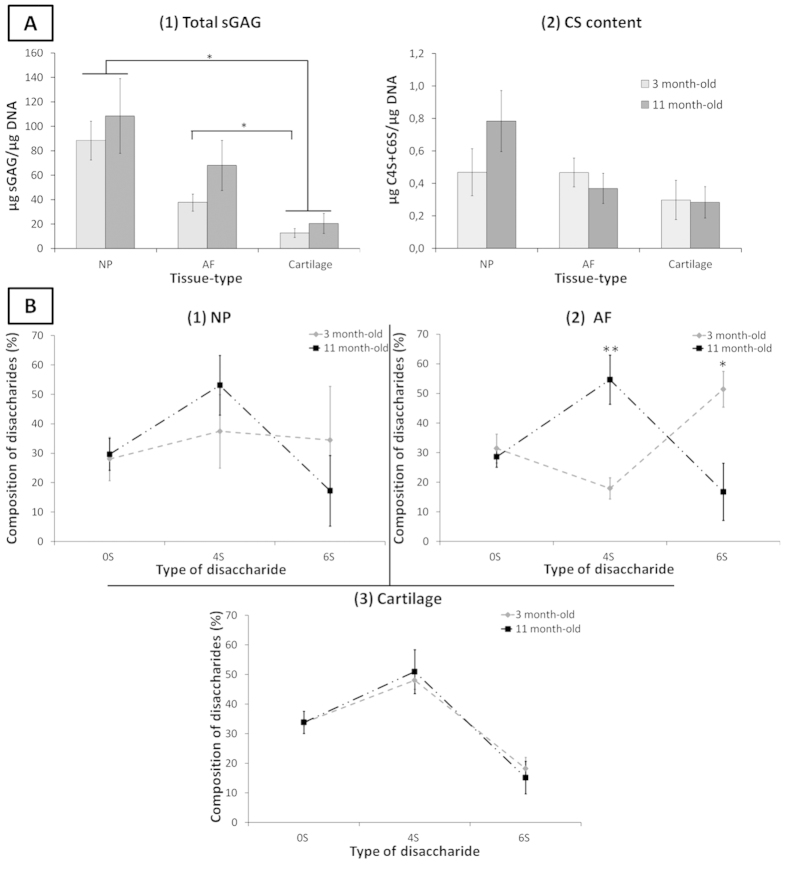
(**A**) Age and tissue-related changes of (1) total sGAG content of ovine IVD quantified by DMMB assay and (2) CS content quantification by HPLC. Percentage of disaccharides C0S, C4S and C6S in three and eleven month-old ovine (1) NP, (2) AF and (3) cartilage tissue. Data were normalised to DNA content and represented as mean ± standard error of the mean (n = 5). *represents significant differences at *p *< 0.05.

DMMB quantification revealed higher sGAG content for all tissue types upon maturity, with the greatest quantity in NP tissue and remarkably lowest quantity in cartilage for both age-groups (Fig. 5A1). The total CS content determined through HPLC analysis was equivalent in immature NP and AF tissue, both greater than immature cartilage. Upon maturity, the total CS content increased in NP tissue, decreased slightly in AF while remaining constant in cartilage (Fig. 5A2). However, no statistically significant differences were observed between age-groups and tissue-types. The proportion of C4S and C6S varied upon maturity with an overall increased in the proportion of C4S and of decrease in C6S in both AF and NP over time (Fig. 5B, Table S2). Cartilage CS composition remained constant upon maturation (Fig. 5B3).

### Distinguishing tissue and cell type and identification of on/off markers

**Figure 6:**
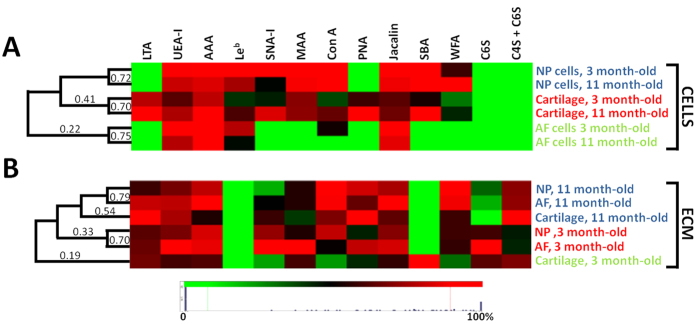
Clustering analysis of **(A)** the average cell glycosylation profile and **(B**) the average ECM glycosylation profile. Data were normalized to maximal individual lectin intensity (100%) (n = 5). Rows and columns denote tissue type and age and lectins, respectively.

Clustering analysis of carbohydrate binding revealed distinct cell and ECM type glycoprofiles. Immature and mature cells from the same tissue were strongly similar (≥70% similarity) (Fig. 6). Chondrocytes and NP cells exhibit a close but distinct phenotype with 41% similar, independent of maturity. In contrast, ECM glycosylation profiles demonstrated ECM was more similar by age rather than by tissue type (Fig. 6) with a high similarity of NP and AF ECM (≥70%). Immature cartilage ECM presented a clear distinct phenotype from all other tissues (19%).

## Discussion

Cellular glycosylation and glycoproteins present a highly transient signature, tightly regulated by biologically active molecules and the cell micro-environment^23^,^27^. Their glycodynamics can dictate the fate of a tissue during development, ageing and pathology^36^. In this work, a library of lectins and carbohydrate-binding antibodies were used to identify spatial and temporal glycosylation changes in IVD and cartilage tissue from immaturity to maturity.

Relative differences in gene and protein expression have been shown between IVD and cartilage tissues^13^,^19^,^34^. However, no on/off markers distinguishing IVD cells have previously been identified through these analyses. Remarkable differences between cell types were revealed here with clear on/off cell type markers identified, which enabled the discrimination of AF and NP cells and chondrocytes (Fig. 6). AF cells were distinguishable from both chondrocytes and NP cells by the absence of binding of the lectins MAA, SNA-I, SBA and WFA. A distinction of chondrocytes from both NP and AF cells were also noticed with the remarkable binding of LTA and PNA on this cell type.

Glycoprofiling demonstrated that chondrocytes and IVD cells were glycophenotypically distinct, independent of age with a close similarity between NP cells and chondrocytes to that of AF cells. These observations correlated with several gene expression analyses which reported similarities between chondrocytes and NP cells^17^,^18^,^37^. However, it is the first time that clear on/off markers were identified that allows to distinguish the three different cell types of the IVD tissue. Bovine and ovine IVDs resemble human IVDs by the occurrence of notochordal cells with a few cells at birth which decrease rapidly with maturity^32^,^38^,^39^. Here, notochordal cells seemed to be absent or at a low number for both age-groups as no cells with notochordal cell morphology were observed in NP tissues (Fig. S1 Supplementary information). No subset cell population was identified by glycoprofiling in NP tissues in the present study. However, it is highly probable that the cell population stained here is a blend of NP cells, notochordal cells and progenitor cells of which NP cells are predominant. The absence of difference in staining indicates a similarity at a glycoprofile level between the three cell types^9^. It is also essential to point out that the differences in glycophenotype identified here will require validation in human tissue^13^.

Distinct ECM profiles of cartilage, NP and AF tissues were identified with different glyco-signature in an age-dependent manner. Closer similarity between NP and AF tissues were shown. The glycosylation alterations play important roles in a variety of biological processes such as tissue development, angiogenesis, inflammation, adhesion, differentiation, cell homing, and cell-cell and cell-ECM interactions^24^,^28^. Fibronectin, a multifunctional glycoprotein, plays a role of connecting molecule between ECM and cells *via* carbohydrate interactions to the cell surface. Cellular fibronectin contains both α-(1→6)-linked Fuc and ⌏-(2→3)-linked sialylation. The fibronectin glycosylation varies depending on tissue and cell types and with pathological conditions such as rheumatoid arthritis^40^. α-(1→6)-linked Fuc, already reported on primary chondrocytes^41^, was observed exclusively on chondrocytes in this study (Figs 1 and 2) which suggests a cartilage-specific tropism for this structure on these cells^28^, potentially related to a role in tissue organisation.

Fibromodulin, another ECM protein, is substituted in articular cartilage with keratan sulfate (KS) and *N*-linked oligosaccharides. This protein has a role in structural maintenance of collagen fibrils, with KS controlling collagen fibril diameter and interfibrillar spacing. Bovine KS is modified with sulfated Gal and GlcNAc, α-(2→3)- and α-(2→6)-linked sialic acid and α-(1→3)-linked fucose, the latter two structures only appearing upon maturity (Figs 1 and 2)^40^. Similar trends were observed in this study, with an increase in α-(2→6)-sialic acid in mature cartilage ECM combined with a decrease in α-(2→3)-linked sialylation while the inverse was observed for α-(2→6)-sialylation in NP and AF ECM, which may be related to tissue organisation and remodelling over development.

Immature NP cells had a higher expression of α-(2→6)-linked sialic acid compared to chondrocytes and expression was inverted upon maturity (Fig. 2). Combined with the overall higher expression of α-(2→6)-linked sialic acid for NP cells compared to chondrocytes, this implies modulated ECM interactions with lectins of similar affinities e.g. siglecs or glycosylated receptors on IVD cells such as integrins and CD44 which can modulate cell-ECM interactions^41^. Rather extraordinarily for differentiated cells which normally have an abundance of complex type *N*-linked oligosaccharides on the cell surface, typically including sialylation^23^, no sialylation was detected on AF cells. Thus AF glycosylation must be either high mannose (Fig. 3) (which evidently disappears upon maturation) and/or Gal-terminated complex and hybrid structures. The interactions with these structures are probably occurring *via* galectins, a family of β-galactoside binding lectins, may be more important for these cells.

High mannose type *N-*linked oligosaccharides were present on all immature cells but not mature AF cells (Fig. 3). Interestingly, a high proportion of surface-expressed high mannose structures is associated with cell differentiation ability^23^,^25^. These structures has also been shown to play a crucial role in initiation and progression of osteoarthritis^42^. Tissue remodelling ability of cartilage and IVD cells may be associated with high mannose structures for IVD cells and chondrocytes, with a decrease in this ability for mature AF cells.

PNA, detected T-antigen, has been shown to be a marker for pre-chondrogenic condensation during development^43^. In agreement to this study, PNA here (Fig. 3) only bound to chondrocytes. Sialylated and possible sulfated T-antigen were observed on NP and AF cells, respectively. One sub-population that may correspond to the AF progenitor cells found in AF tissue^44^ which exhibit a different glycosylation profile than that of AF cells was detected as GS-I-B4 binding (Fig. S3). Terminal GalNAc was detected in all ECM tissues for both age-groups. As WFA tolerates the presence of sulfation, sulfated GalNAc as found in CS GAGs were most likely more predominant in all ECM types, with less sulfation the immature cartilage ECM. This is confirmed with a higher binding with SBA (Fig. 4) which was drastically decreased upon maturity. Interestingly, terminal GalNAc was detected on both NP cells and chondrocytes but not at all on AF cells. This reinforces the idea of a different set of interactions for AF cells in comparison NP cells and chondrocytes.

Sulfated GAG content (Fig. 5) is highly related to tissue hydration and hence plays an essential role in the organisation of the ECM and its water content^45^. No significant differences in overall CS content were observed in this study with maturation. However, there were clear differences in sulfation pattern between the AF and NP tissues and age-groups. Both the NP and AF sulfation pattern changed considerably over time, with an inversion of the sulfation type from predominantly C6S at immaturity, consistent with previous studies in embryonic IVD^6^, to predominance of C4S at maturity. This switch might be related to a change of the mechanical loads on the tissue upon maturation, shown to affect CS sulfation in cartilage^46^. In addition, CS fine structure and sulfation pattern were shown to be cell specific (biomarkers for progenitor cells in cartilage)^47^, to be modified under pathological conditions in cartilage^46^,^48^, and to alter cell surface glycosylation in neuronal cells when changes in GAG composition of the ECM^26^. In immature tissues, blood vessels were observed (Fig. S1) in the outer immature AF, while no vessels were noted in mature tissue as expected. The higher percentage of C4S compared to C6S observed at maturity may relate to a greater ability of the tissue to inhibit nerve and blood vessel growth in the IVD tissue^2^,^49^,^50^.

In conclusion, tissue- and cell-specific glycosylation was identified which was altered spatially and temporally. Further analyses are required to correlate the changes observed here to the changing anatomical and mechanical demands on the tissues during development and growth and/or remodelling activities. Specific on/off markers were identified which allow distinction between NP and AF cells and will aid specific cell type isolation and verification of MSC differentiation. A distinct switch in CS sulfation pattern was identified in AF and NP ECM upon maturation most probably related to the tissue function. This study sheds light on cell-cell and cell-ECM phenomena crucial to the design of new regenerative approaches for IVD and cartilage therapies.

## Materials and Methods

### Tissue collection and preparation

Three and 11 month-old ovine spine and articular cartilage (AC) from the hip joint were collected from a local abattoir directly after sacrifice (n=5). Soft tissues surrounding IVD (L4-L5) and cartilage were removed and both tissues were harvested. Half of each tissue (transversal cut) was fixed with 4% paraformaldehyde. After three washes with PBS, tissues were infiltrated overnight with 20% sucrose, flash-frozen in liquid nitrogen-cooled isopentane and 5 μm sections were cut horizontally on a Leica CM 1850 cryostat. Sections were collected on Superfrost^®^ Plus slides (ThermoFisher Scientific Inc.) and stored at −20 °C until use. The remaining NP, AF and AC tissues were separated and digested with proteinase K (0.5 mg/mL) at 56 °C overnight.

### Biochemical assays

Sulfated GAG (sGAG) tissue content was quantified by dimethylmethylene blue (DMMB) assay at pH 3.5 using chondroitin 4-sulfate (C4S) from bovine trachea (Sigma-Aldrich^®^) as standard and measuring absorbance at 535 nm. DNA quantification was performed using the Quanti-iT^TM^ Picogreen^®^ assay per manufacturer instructions (Life Technologies^TM^).

### Lectin and immuno-histochemistry

All washes were three times for 5 minutes each, all steps performed at room temperature in a humidity chamber unless otherwise stated. Three slides were used for each lectin or antibody incubation. For lectin histochemistry, slides were washed with Tris-buffered saline supplemented with Ca^2+^ and Mg^2+^ (TBS; 20 mM Tris-HCl, 100 mM NaCl, 1 mM CaCl_2_, 1 mM MgCl_2_, pH 7.2) with 0.05% Triton X-100 (TBS-T) and then blocked with 2% periodate-treated BSA (Sigma-Aldrich^®^) in TBS for 1 hour. Sections were washed then incubated with 11different fluorescein isothiocyanate (FITC)- or tetramethylrhodamine isothiocyanate (TRITC)-conjugated lectins (EY Labs Inc.) in TBS for 1 hour (Table S1). Inhibitory controls were carried out in parallel to verify lectin binding specificity by pre- (for 1 hour) and co-incubating lectins in 100 mM of the appropriate haptenic sugar in TBS (Table S1). Sections were washed five times with TBS-T and counterstained with 4′6-diamidino-2-phenylindole dihydrochloride (DAPI; 2⌏g/ml) for 20 minutes. The slides were washed in TBS-T before mounting the coverslip with ProLong^®^ Gold antifade (Life Technologies^TM^). Inhibition by the appropriate haptenic sugar was observed for all lectins used (data not shown).

For all immunohistochemistry, sections were blocked with 5% goat serum in PBS for 1 hour before overnight incubation at 4 °C with the primary antibodies, with negative control sections incubated with PBS, followed by five washes in PBS with 0.05% Tween 20 (PBS-T). The three primary antibodies selected recognised chondroitin 6-sulfate (C6S), C6S and C4S, and Lewis^b^ (Le^b^) (Abcam^®^) (Table S1). For the anti-C6S antibody, antigen retrieval was first performed with 0.25U/ml of chondroitinase ABC (ChABC, Sigma-Aldrich) in 50 mM Tris-HCl, 60 mM sodium acetate, pH 8.0 for 30 minutes followed by PBS washes. Sections were incubated with Alexa Fluor^®^ 488-conjugated donkey anti-mouse (Life Technologies^TM^) at 1/1,000 dilution in PBS for 1 hour then washed five times with PBS-T before counterstaining with DAPI (2⌏g/ml in PBS) for 10 minutes. The sections were washed with PBS-T before mounting the coverslip as above.

All slides were cured at 4 °C in the dark for 1 day before imaging with an Olympus IX81 inverted epifluorescent microscope (five images per section).

### Image analysis and quantification

Image fluorescence intensity was quantified using ImageJ software (National Institutes of Health, http://rsbweb.nih.gov/ij/). From each image, five cells and five ECM areas were surrounded, fluorescence intensity measured and intensity values normalised by surface area. For each image, the mean of five values for cells and ECM was taken. The average from five images per section represented the quantification of one tissue section per animal. Average fluorescence intensity quantification from five animals with standard error of the mean was presented. For the clustering analysis, data were normalised to maximal individual lectin or antibody intensity across sections and the clustering analysis was performed in Hierarchical Clustering Explorer 3.0 (National Institutes of Health, USA) without additional normalisation, with complete linkage and Euclidean distance. Results were represented using a heat map.

### Chondroitin sulfate analysis

Proteinase K-digested NP, AF or cartilage tissues was filtered through an Amicon Ultra 3kDa molecular weight cut off (MWCO) centrifugal filter (Millipore) with 100μL HPLC-grade water and the retentate was digested with 100mU ChABC 50 mM Tris-HCl, 60 mM sodium acetate, pH 8.0 for 3 hours at 37 °C. The digested mixture was filtered through a 3kDa MWCO centrifugal filter, filtrate dried in a vacuum centrifuge and stored at −20 °C.

For analysis, the unsaturated chondroitin sulfate disaccharide (⌏di-0S, ⌏di-4S and ⌏di-6S) content determined as previously described^51^, with minor modifications. In brief, each sample was separated on an Ultratech 5ODS C18 (250 × 4.6 mm) (HPLC Technology Inc.) or a Synergi^TM^ column (250 × 4.6 mm, 4 μm, 80Å) (Phenomenex Inc.) at 25 °C on a Waters Alliance 2695 instrument. Absorbance was monitored at 232 nm using a Waters 2489 detector. Standard curves of Δdi-0S, Δdi-4S and Δdi-6S (Dextra Labs Ltd.) were generated and quantification data were presented as μg of disaccharide per μg of DNA.

### Statistical analysis

Statistical analysis was performed using GraphPad Prism^®^, v.5 (USA). Data were compared using ANOVA followed by Tukey comparison or non-parametric Kruskal-Wallis test followed by Dunn’s comparison test when the population followed a normal and a non-normal distribution (D’Agostino and Pearson omnibus normality test), respectively. Values were considered significantly different with *p *< 0.05.

## Additional Information

**How to cite this article**: Collin, E. C. *et al.* Unique glycosignature for intervertebral disc and articular cartilage cells and tissues in immaturity and maturity. *Sci. Rep.* **6**, 23062; doi: 10.1038/srep23062 (2016).

## Supplementary Material

Supplementary Information
