# Supplementary material for: Unique glycosignature for intervertebral disc and articular cartilage cells and tissues in immaturity and maturity
Source: Sci Rep. 2016 Mar 11;6:23062. doi: 10.1038/srep23062 (PMC4786852; doi:10.1038/srep23062)
Supplement: Supplementary Information [file srep23062-s1.pdf]

Supplementary information

Title: Unique glycosignature for intervertebral disc and articular cartilage cells and tissues in immaturity and maturity

E.C. Collin[1,†], M. Kilcoyne[2,3,†], S.J. White[3], S. Grad[4], M. Alini[4], L. Joshi[1,3], A.S. Pandit[1,*]

[1] Center for Research in Medical Devices (CÚRAM), National University of Ireland Galway, Ireland

[2] Microbiology, School of Natural Sciences, National University of Ireland Galway, Ireland

[3] Glycoscience Group, National University of Ireland Galway, Ireland

[4] AO Research Institute, Davos, Switzerland

[*]Corresponding authors

[†] These authors have contributed equally.

Lectin/antibody	Abbreviation	Binding specificity	Concentration (mg/mL)	Inhibitory glycan
Lotus tetragonolobus agglutinin	LTA	Fuc-α-(1→3), Fuc-α-(1→6), Fuc-α-(1→2)	15	100mM Fuc
Ulex europaeus agglutinin I	UEA-I	Fuc-α-(1→2)	10	100mM Fuc
Aleuria aurantia agglutinin	AAA	Fuc-α-(1→6), Fuc-α-(1→3)	20	100mM Fuc
Anti-Lewis^b antibody	Le^b	Fuc-α-(1→2)-Gal-β-(1→3)-[Fuc-α-(1→4)]GlcNAc	$1.25x10^{-3}$	---
Sambucus nigra agglutinin I	SNA-I	Neu-α-(2→6)-GalNAc > Lac, GalNAc > Gal	20	100mM Lac
Maackia amurensis agglutinin	MAA	Neu-α-(2→3)-GalNAc, Gal-3S > Lac	20	100mM Lac
Griffonia simplicifolia isolectin	GS-I-B_4	Terminal α-Gal	10	100mM Gal
Concanavalin A	Con A	a-Man > a-Glc > a-GlcNAc, complex biantennary structures	20	100mM Man
Peanut agglutinin	PNA	Gal (Gal-β-(1→3)-GalNAc (T-antigen) > GalNAc > Lac > Gal, terminal β-Gal)	10	100mM Gal
Artocarpus integrifolia agglutinin	Jacalin (AIA)	Gal, Gal-β-(1→3)-GalNAc (T-antigen), Gal-α-(1→6), sialylation independent	10	100mM Gal
Soya bean agglutinin	SBA	GalNAc > Gal	10	100mM GalNAc
Wisteria floribunda agglutinin	WFA	GalNAc, GalNAc-α-(1→6)-Gal > GalNAc-α-(1→3)-GalNAc (Forsmann antigen) > GalNAc >> Lac > Gal, GlcA-α-(1→3)-GalNAc	10	100mM GalNAc
Anti-chondroitin-4- and -6-sulfate antibody (CS-56 clone)	CS-56	GlcA-α-(1→3)-GalNAc-4S, GlcA-α-(1→3)-GalNAc-6S	1/200 dilution	---
Anti-chondroitin-6-sulfate antibody	C6S	GlcA-α-(1→3)-GalNAc-6S	1/200 dilution	---

Table S1. Lectins and antibodies used for tissue histochemistry, their specificities, concentrations and inhibitory carbohydrates.

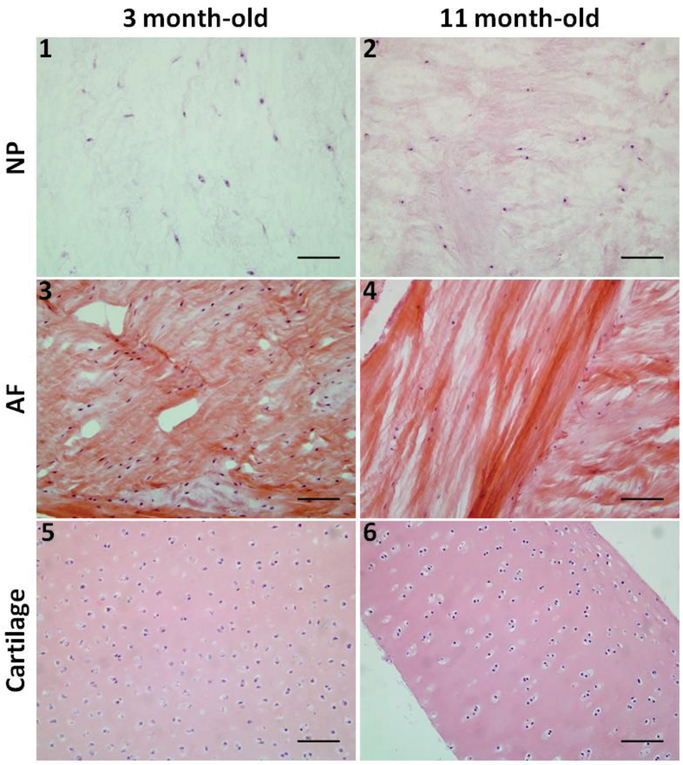

Figure S1. Representative images of hematoxylin and eosin stained ovine NP **(1-2),** AF **(3-4)** and cartilage tissue **(5-6)** at 3 and 11 months. ECM proteins and nuclei are stained in pink/red and purple/blue, respectively. Scale bar = 100μm.

Table S2. Quantity of C0S, C4S and C6S disaccharides (µg/ µg of DNA) in 3 and 11 month ovine NP, AF and cartilage tissues. Data were normalized to DNA content and presented as mean ± standard error of the mean (n=5). No significant difference was seen between the different groups (p<0.05).

Age (months), tissue	***C0S***	***C4S***	***C6S***	***Total***
**3, NP**	0.14±0.03	0.19±0.03	0.16±0.15	0.47±0.11
**11, NP**	0.25±0.08	0.43±0.14	0.04±0.01	0.85±0.23
**3, AF**	0.13±0.02	0.10±0.01	0.24±0.09	0.47±0.09
**11, AF**	0.10±0.03	0.21±0.07	0.03±0.01	0.37±0.10
**3, cartilage**	0.10±0.04	0.15±0.06	0.05±0.02	0.30±0.12
**11, cartilage**	0.10±0.04	0.14±0.05	0.04±0.02	0.29±0.10

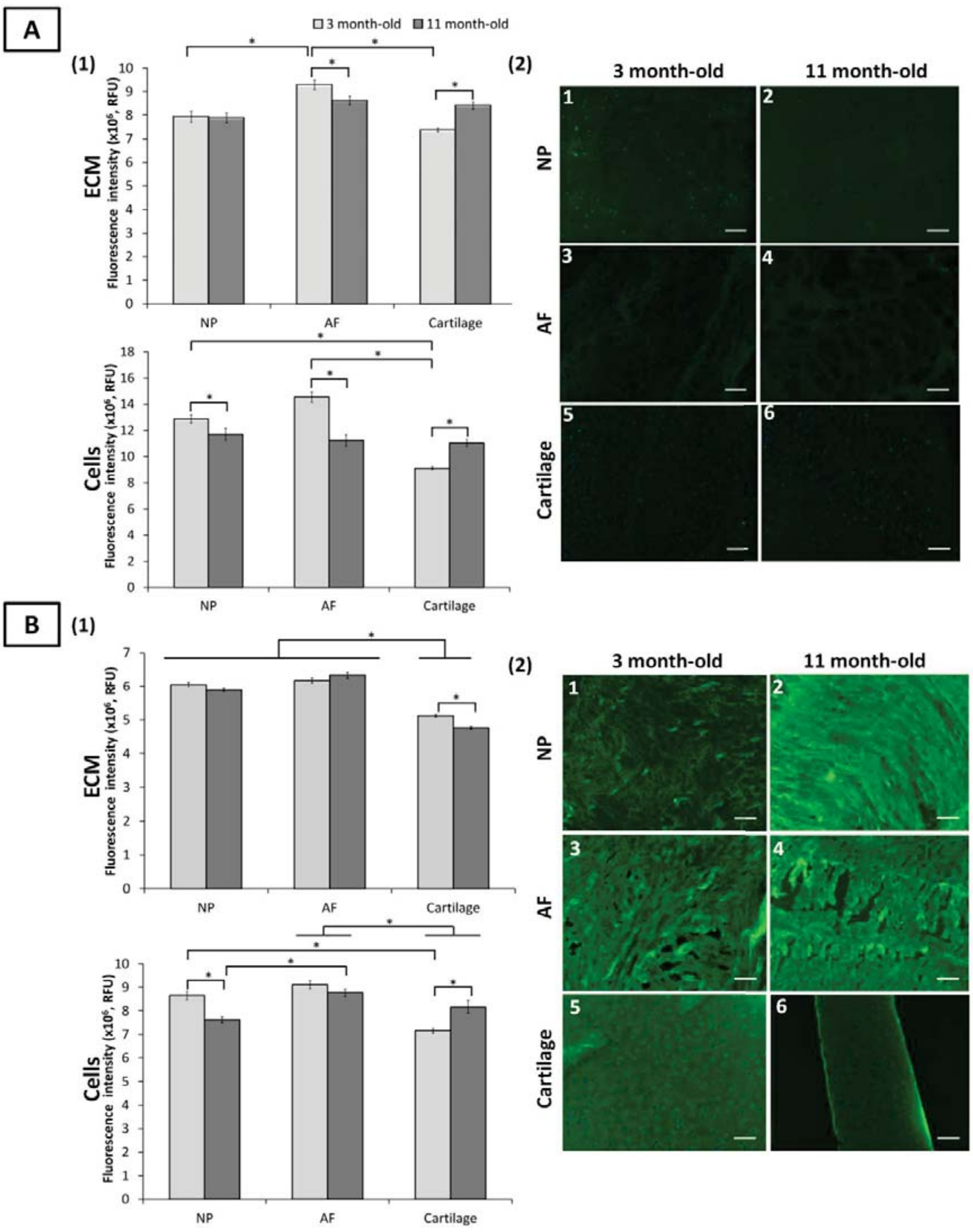

Figure S2. Fucosylated motifs detected by UEA-I and AAA lectin staining in ovine NP, AF and cartilage tissues at three and 11 months. Quantification of UEA-I (A1) and AAA (B1) lectins binding to the ECM and onto the cells of NP, AF and cartilage tissues. Data were normalised to surface area and represented as mean ± standard error of the mean (n = 5). * denotes significant differences between the different groups at $p<0.05$. **Representative fluorescent images of the stained motifs detected by UEA-I (A2) and AAA (B2) lectins binding in NP, AF, and cartilage tissues.** Fucosylated motifs and nuclei are stained in green and blue, respectively. Scale bar = 100µm.

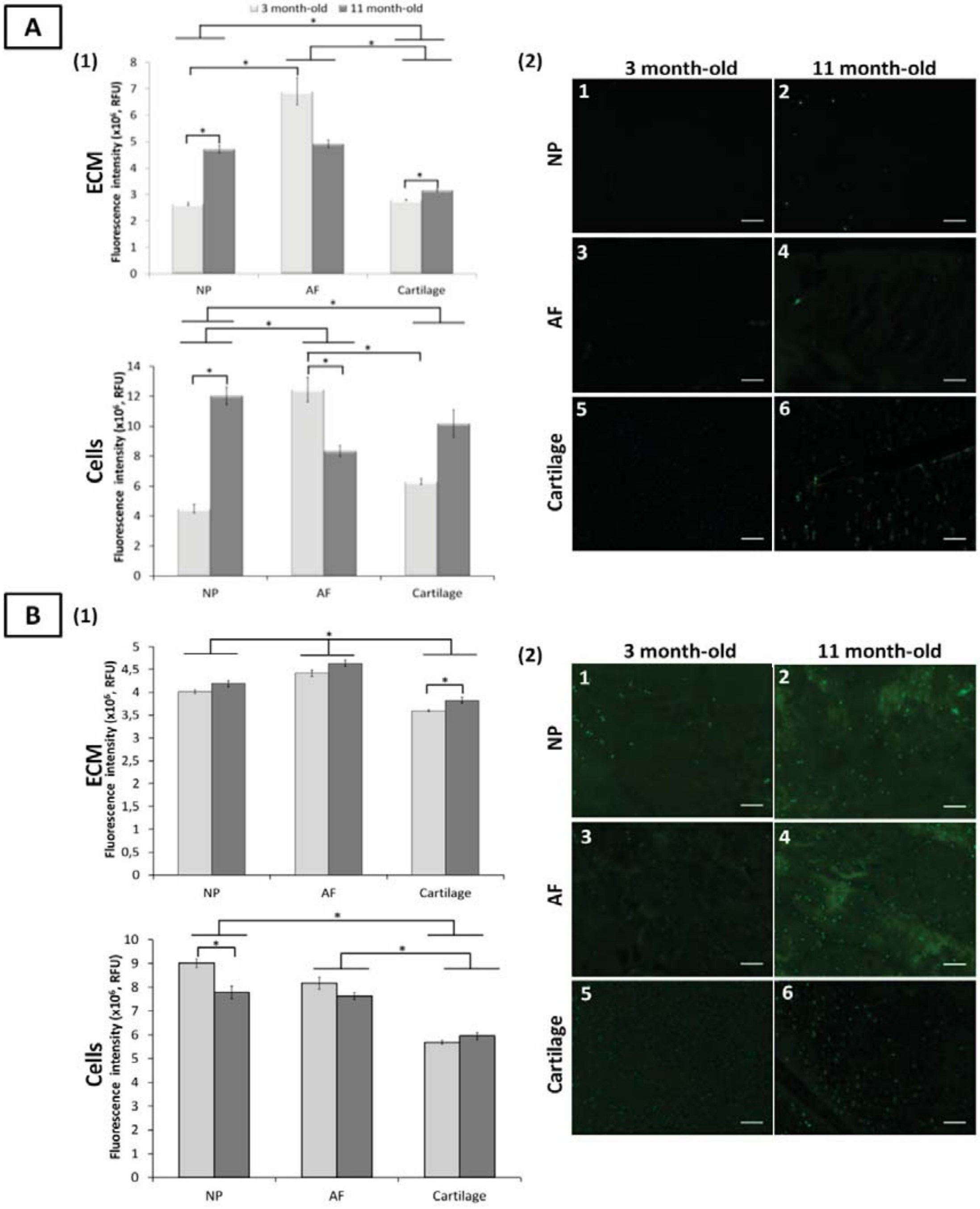

Figure S3. Terminal α-Gal motifs detected by GS-I-B_4 lectin staining and galacosylated motifs detected jacalin lectin staining of ovine NP, AF and cartilage tissues at three and 11 months. Quantification of GS-I-B_4 (A1) and jacalin (B1) lectins binding to the ECM and onto the cells of NP, AF and cartilage tissues. Data were normalised to surface area and represented as mean ± standard error of the mean (n = 5). * denotes significant differences between the different groups at p<0.05. **Representative fluorescent images of the stained motifs detected by GS-I-B_4 (A2) and jacalin (B2) lectins binding in NP, AF, and cartilage tissues.** Motifs and nuclei are stained in green and blue, respectively. Scale bar = 100μm.

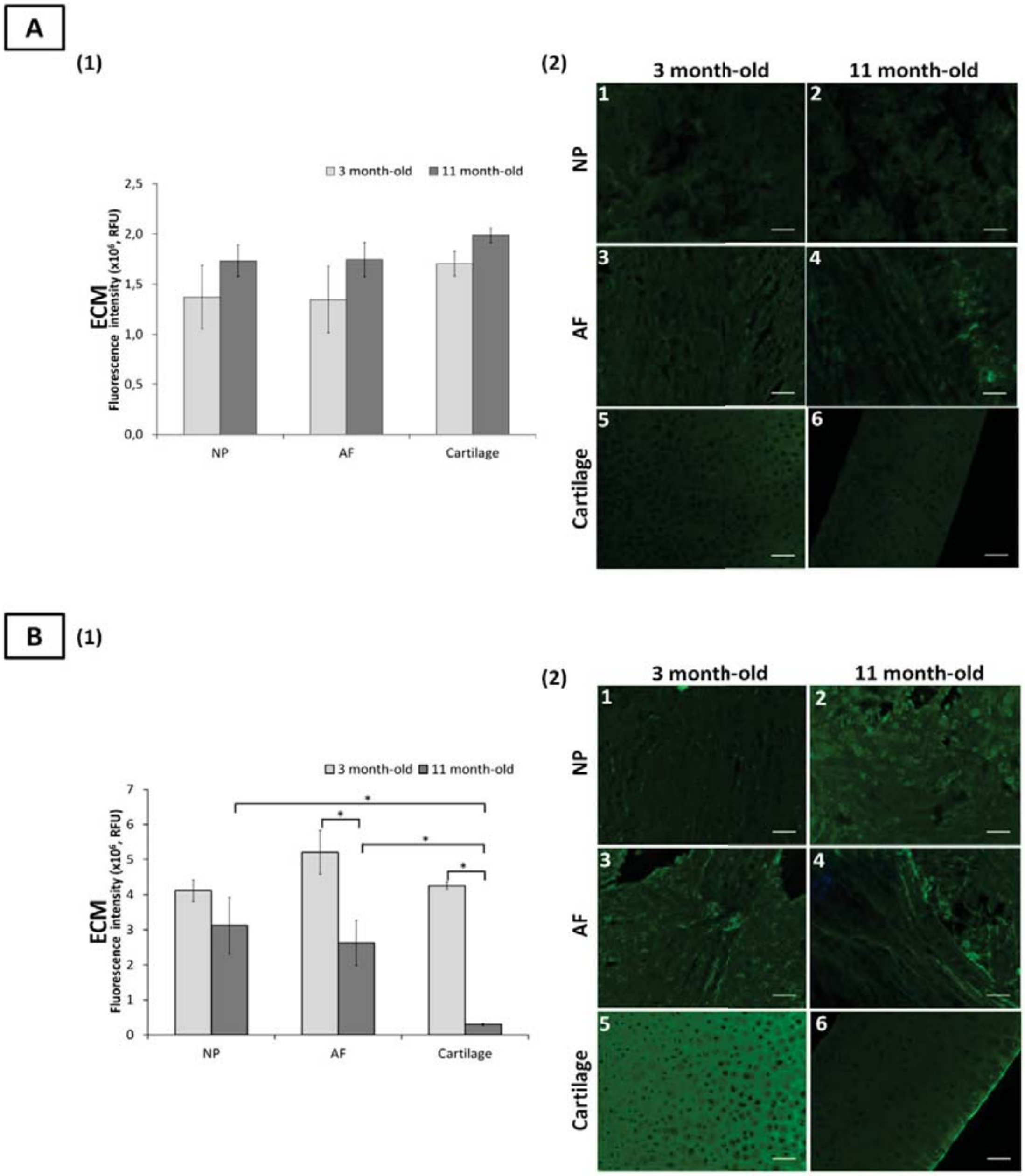

Figure S4. Chondroitin sulfate detected by CS-56 (C4S + C6S) and C6S antibody staining in ovine NP, AF and cartilage tissues at three and 11 months. Quantification of CS-56 (C4S + C6S) (A1) and C6S antibody (B1) binding to the ECM and onto the cells of NP, AF and cartilage tissues. Data were normalised to surface area and represented as mean ± standard error of the mean (n = 5). * denotes significant differences between the different groups at $p<0.05$. **Representative fluorescent images of the stained motifs detected by CS-56 (C4S + C6S) (A2) and C6S antibody (B2) binding in NP, AF, and cartilage tissues.** Motifs and nuclei are stained in green and blue, respectively. Scale bar = 100µm.
